# Supplementary material for: Stable vortices in the anomalous metallic state observed on monoatomic-layer superconductors
Source: Sci Adv. 2026 Mar 20;12(12):eadu9610. doi: 10.1126/sciadv.adu9610 (PMC13004047; doi:10.1126/sciadv.adu9610)
Supplement: Supplementary file 1 — Supplementary Text S1 to S7 Figs. S1 to S8 References [file sciadv.adu9610_sm.pdf]

Science Advances
AAAS

Supplementary Materials for

Stable vortices in the anomalous metallic state observed on monoatomic-layer superconductors

Yudai Sato *et al.*

Corresponding author: Yudai Sato, yudai.sato@lmu.de

Sci. Adv. **12**, eadu9610 (2026)
DOI: 10.1126/sciadv.adu9610

This PDF file includes:

Supplementary Text S1 to S7
Figs. S1 to S8
References

Supplementary text 1. Crystallographic orientation of the tilting of the vicinal substrates

The vicinal substrates used in this study are tilted toward the $[\bar{1}\bar{1}2]$ direction. To confirm this, we obtained several STM images of the 1.1°-tilted vicinal substrates covered with a 7 × 7 surface-reconstructed structure, as presented below. It is known that the faulted half of the 7 × 7 structure, which has a triangular shape pointing in the $[\bar{1}\bar{1}2]$ direction of the substrate, is imaged brightly in the empty-state images (Fig. S1C), the images presented in Fig. S1 confirm that the substrate is tilted toward the $[\bar{1}\bar{1}2]$ direction.

Supplementary text 2. Set-up of transport measurements

In order to measure the electrical conductance of the Pb-adsorbed SIC samples, a four-terminal pattern with the probed area of 1.2 × 0.3 mm^2 (shown in Fig. S2(A)) was covered with a shadow mask, and the rest of the Pb layer was removed by Ar^+ sputtering.

Resistance was calculated as the measured voltage divided by the DC bias current I_S (1 μA). We confirmed that our I_S is sufficiently low to avoid the enhancement of resistance with increase in *I*s in 2D system (70) as following:

- I_S is much lower than the critical current ~20 uA, as indicated by the voltage drop in Fig. S2(B, C). Indeed, Fig. S2(D, E) shows resistance is zero around 1 uA.
- As shown in Fig. S2, the width of the current path in our transport measurements is 0.3 mm. Therefore, 2D current density J_S of our system is given by I_S/(0.3 mm) = 3.3 mA/m, which is lower than that of a-MoGe thin film (72), where no

I_S dependence of resistance is observed.

- We have also used the same transport setup in previous measurements on similar samples (78). The Fig. 5B shows zero resistance under magnetic fields up to 100 mT, using the same I_S as in the present study. The sample was √7x√3-In on Si(111), a well-known atomic-layer superconductor formed on Si(111) as well as our target Pb-SIC on Si(111). Notably, the gap size and the superconducting transition temperature T_c √7x√3-In (0.57 meV and 3.18 K) (9) are comparable to those of Pb-SIC (0.33 meV and 1.53 K).

Supplementary text 3. Transport and STM results at zero magnetic field

In the case of two-dimensional superconductors, the conductivity is enhanced near and above the critical temperature (T_c) because of thermal fluctuations, which is known as paraconductivity. The temperature (T)- dependent sheet resistance just above T_c is written as the inverse of the paraconductivity:

$$R_{\text{sheet}} = \frac{1}{\frac{1}{R_{\text{N}}(T)} + \frac{e^2}{16\hbar}\left(\frac{1}{\tau} + \frac{2}{\tau - \delta}\ln\left(\frac{\tau}{\delta}\right)\right)}\ , \qquad (S1)$$

where $\tau = T/T_c$ and δ is the pair-breaking parameter. In the denominator of the right side of the equation, the second term is Aslamasov-Larkin (AL) term and the third term is the Maki-Thompson (MT) term. $R_N(T)$ of the first term is the normal resistance. It is difficult to define $R_N(T)$ because it exhibits temperature dependence, such as the Altshuler-Aronov effect induced by disorder (63, 64). Here, we used the linear expression $R_N'(T) = aT + R_N'(0\ \text{K})$, where a is a temperature coefficient. The results of fitting with the parameters T_c and δ are shown in Fig. S3 (A and B) (red curve). We obtained $T_{c,\text{flat}} = 1.53$ K, $\delta_{\text{flat}} = 0.23$, $a_{\text{flat}} = -11.8$, $R_{N,\text{flat}}'(0\ \text{K}) = 6.13 \times 10^2\ \Omega$ for the flat

sample and $T_{\mathrm{c,vicinal}}$(0 mT) = 1.21 K, $\delta_{\mathrm{vicinal}}$ = 0.26 and a_{vicinal} = -38.7, $R_{\mathrm{N,vicinal}}'$(0 K) = 1.20 ×10^3 Ω for the 1.1°-tilted vicinal sample.

The finite resistance below T_{c} is due to the Berezinskii-Kosterlitz-Thouless (BKT) transition (60, 61). Whereas the long-range order of a superconducting order parameter is not allowed at finite temperatures for 2D superconductors according to the Mermin-Wagner theorem (79, 80), superconducting properties are realized below the BKT transition temperature T_{BKT}, where vortex-antivortex pairs are bound and a quasi-long-range order (topological order) of the phase of the superconducting order parameter is formed. Temperature-dependent sheet resistance due to the BKT transition is described below (81)

$$R_{\mathrm{sheet}} = c\left[-2b\left(\frac{T_{\mathrm{c}} - T_{\mathrm{BKT}}}{T - T_{\mathrm{BKT}}}\right)^{-\frac{1}{2}}\right] \qquad (S2)$$

where b and c are fitting parameters. From the fitting we obtained T_{BKT} = 1.29 K, b = 1.16 and c = 3.31×10^3 Ω for the flat sample, and T_{BKT} = 0.83 K, b = 4.13 and c = 1.71×10^6 Ω for the vicinal sample. The fitting results are shown by the blue curves in Fig. S3 (A and B).

In dirty limit, the relation $1 - T_{\mathrm{BKT}}/T_{\mathrm{c}} \approx 0.2R_{\mathrm{sheet}}/(\hbar/e^2)$ is expected (82), which indicates that T_{BKT} decreases with increasing R_{S}, reflecting enhanced phase fluctuations due to disorder. By using this equation, we can obtain 1.50 K for the flat sample and 1.15 K for the vicinal sample as T_{BKT}, which are larger than T_{BKT} from Eq. (S2). The fitting with Eq. S2 experimentally captures the broadness of the resistance drop below T_{C}; lower T_{BKT} corresponds to a broader transition. This suggests that the transition we observed is broader than what would be expected in the above relation. Therefore, the discrepancy between the two methods implies that our system experiences stronger

phase fluctuations than predicted solely by R_s. A similar trend has also been reported (5).

To evaluate the shape of the zero-bias anomaly (ZBA) shown in Fig. 1 (C and D) in the main text in detail, we took dI/dV spectra in a wider range of bias voltages (±20 meV) and fitted the spectra with $V^{-\alpha}$, where α is the Altsuler-Aronov exponent and is the degree of disorder. As shown in Fig. S3 (C and D), we found that α obtained on the vicinal sample (0.133) is larger than that obtained on the flat sample (0.091). This is consistent with the fact that the ZBA gap deepens with increasing degree of disorder.

Supplementary text 4. Magnetic field dependence of the extracted activation energy *U*(*B*)

Figure S5 (A and B) show the magnetic field dependence of the extracted activation energy $U(B)$ determined by the thermally activated behavior $R = R'\exp(-U(B)/k_BT)$ presented in the Arrhenius plots of $R_s(T)$ of the flat sample (Fig. S4C) and vicinal (Fig. 2B) samples, respectively. The insets show the relationship between $U(B)/k_BT_c$ and ln R'. $U(B)$ and R' are well described by the equations $U(B) \propto \ln(B_0/B)$ and $\ln R' = U(B)/k_BT_c$ +*const*, suggesting that it is due to the thermally assisted collective vortex creep in 2D superconductors (83). Interestingly, we have strong curvatures in higher magnetic fields in Fig. S5(A, B) and similar one has also been observed in weakly disordered system (4).

Supplementary text 5. Critical magnetic field B_{c2} estimated from the Ullah Dorsey (UD) scaling theory

The Ullah Dorsey (UD) scaling theory (73), which is based on the time-dependent Ginzburg Landau (GL) equation under the Hartree approximation, describes the mean field upper critical magnetic field of superconductors that exhibit a vortex state and various fluctuation effects, such as a two-dimensional superconducting system. The UD rule is expressed as follows:

$$G_{\mathrm{fl}}(T)\sqrt{\frac{B}{T}} = \mathrm{F}\left(\frac{T - T_{\mathrm{c}}^{\mathrm{MF}}(B)}{\sqrt{TB}}\right). \qquad (S3)$$

where $G_{\mathrm{fl}}(T) = 1/R_{\mathrm{S}}(T) - 1/R_{\mathrm{N}}$. In the analysis described below, the resistance measured at 500 mT was used as the normal-state resistance R_{N}. $T_{\mathrm{c}}^{\mathrm{MF}}(B)$ is the mean field transition temperature under magnetic field B. F(x) is a scaling function proportional to $-x$ ($x \ll 0$) and x^{-s} ($x \gg 0$), where $s = 1$ in the case of two-dimensional superconductors. Figures S5 (C and D) show the results of UD scaling using the data in Fig. 2A in the main text and Fig. S4B. The extracted $T_{\mathrm{c}}^{\mathrm{MF}}(B)$ is plotted in Fig. 2 (E and F) at temperatures higher than $T_{\mathrm{c}}(B = 0)/2$, where UD scaling theory is applicable.

Supplementary text 6. The temperature dependence of B_{c2} fitted by the Wertharmer-Helfand-Hohenberg (WHH) theory

The upper critical magnetic field $B_{c2}(T)$ is given by $\Phi_0/2\pi\xi_{\mathrm{GL}}(T)^2$ when only the orbital pair breaking contributes to the breaking of the superconductivity. However, when the paramagnetic effect and/or spin-orbit scattering also contribute, the upper critical magnetic field deviates from $\Phi_0/2\pi\xi_{\mathrm{GL}}^2$. WHH theory (74) is a comprehensive theory describing the upper critical field, including all three effects. In this theory, the Maki parameter α indicates the contribution of the paramagnetic effect, and λ_{SO} is the strength

of spin-orbit scattering. We found that our results for $B_{c2}(T)$ can be fitted well with the parameters $\alpha = 0$ and $\lambda = 0$, as shown in Fig. 2 (A and B) in the main text, indicating that superconductivity is dominantly broken by orbital pair breaking.

Supplementary text 7. Histogram analysis of ZBC maps

To quantitatively describe the evolution of the vortex distribution and its behavior, we obtained a histogram of the zero-bias conductance (ZBC) from its maps. Figure S7A shows the ZBC histogram of the 1.1°-vicinal sample under zero magnetic field (Fig. 3B), where there are no vortices. The histogram shape can be fitted using a normal distribution function. Under a magnetic field of 30 mT, a vortex is observed in the ZBC map (Fig. 3C), and the ZBC histogram has a single peak that can be fitted with a normal distribution function with an additional tail on the high-ZBC side, as shown in Fig. S7B. The tail is caused by the presence of vortices. By further increasing the magnetic field, the peak position μ, which corresponds to the averaged ZBC outside the vortex cores, gradually increased. At B = 240 mT (Fig. S7D), the shape of the histogram can be fitted by a single normal distribution function, which indicates that the entire area is covered with vortices. The peak position further increases with the magnetic field until B^*, where the pseudogap disappears. We performed the same analysis for the flat sample, and a plot of the peak position vs. magnetic field for the flat and 1.1°-vicinal samples is shown in Figs. 4 (A and B), respectively.

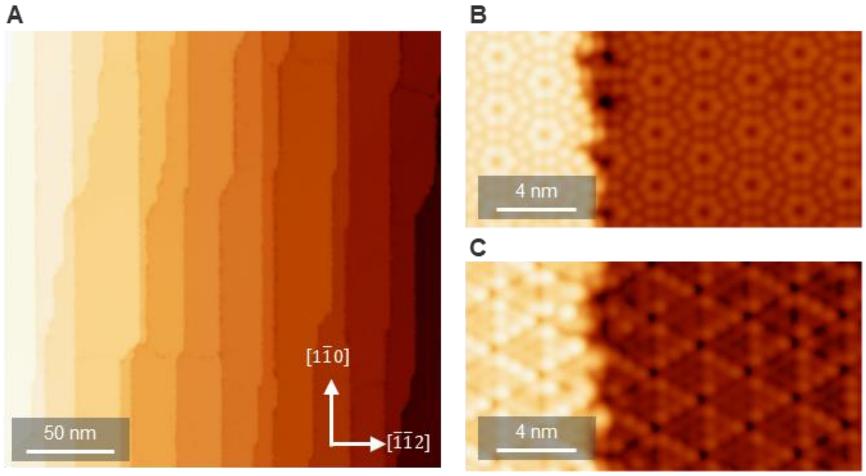

Fig. S1 Crystallographic orientation of the tilting of the vicinal substrates. (**A**) STM image of a 1.1°tilted vicinal Si(111) surface showing a 7 × 7 structure. (V_S = 1.0 V, I_T = 100 pA). (**B** and **C**) zoomed images taken with (**B**) V_S = 1.0 V, (**C**) V_S = -1.4 V, respectively.

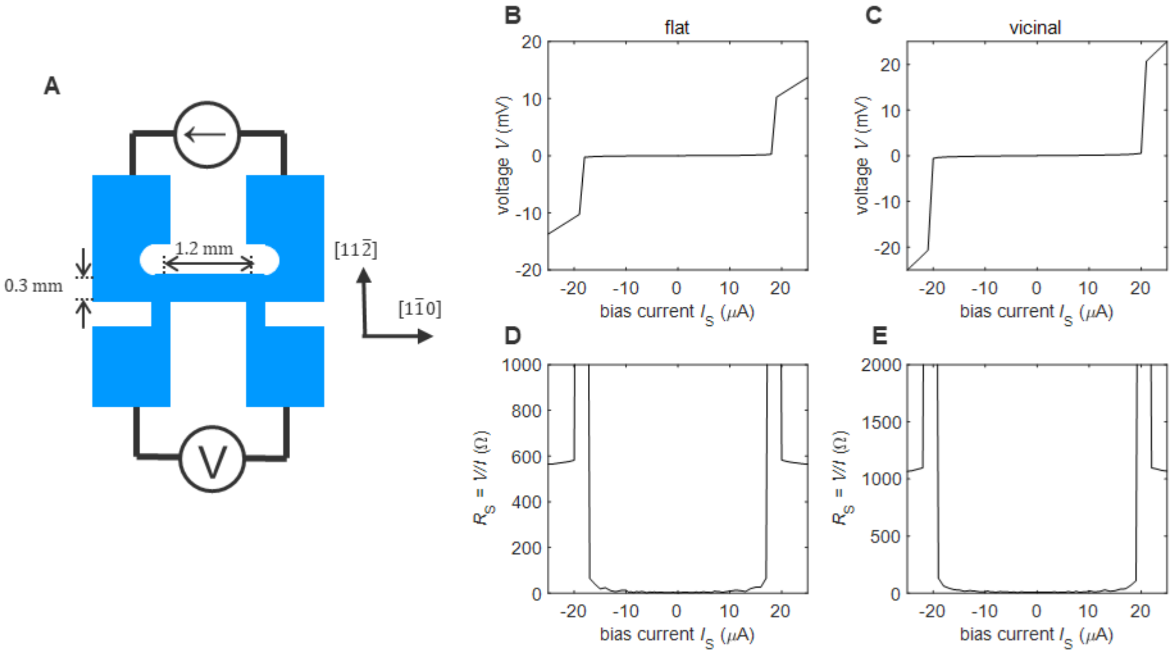

Fig. S2 Set-up of transport measurements. (**A**) Schematic of the electrode configuration for electrical transport measurements. (**B** and **C**) V-I curves for (**B**) the flat and (**C**) the vicinal samples taken at T = 0.4 K and B = 0 T, respectively. (**D** and **E**) $R_S = V/I_S$ as a function of I_S calculated by using (B, C).

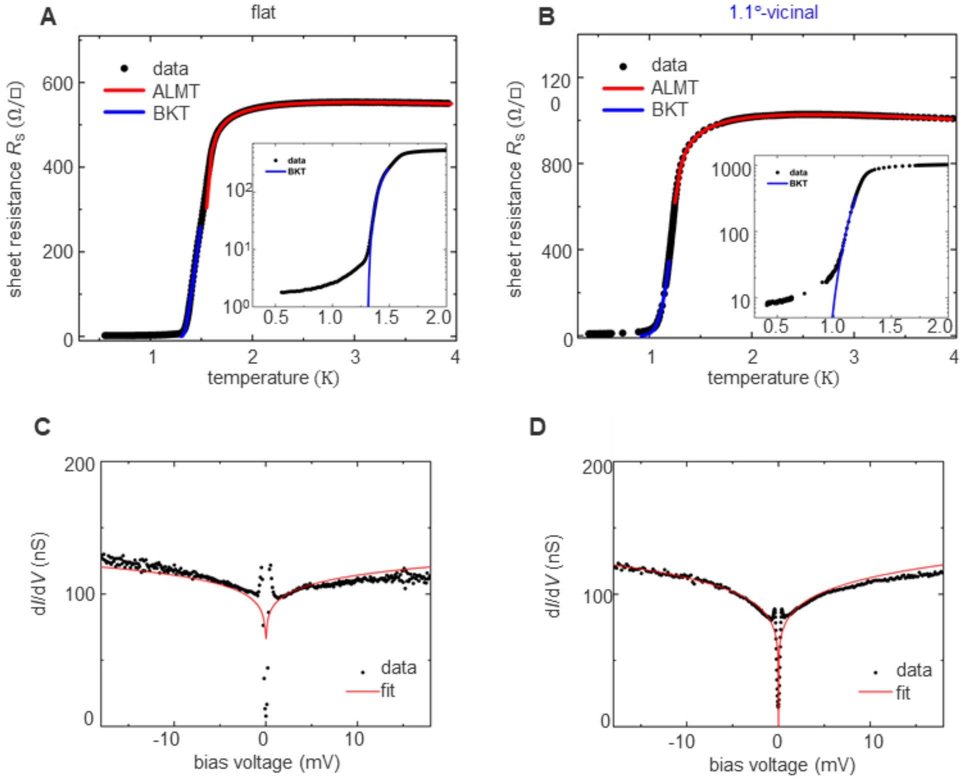

Fig. S3 Transport and STM results of flat and vicinal samples at zero magnetic field. (**A** and **B**) R_S-T curves with fitting results for the flat and vicinal samples, respectively. The red curves show the fitting curves of the paramagnetic conductivity using Eq. (S1). The blue curves show the fitting curves of BKT theory using Eq. (S2). The inset shows a log plot of the sheet resistance. (**C** and **D**) Tunneling conductance dI/dV spectra of the flat and vicinal samples, respectively (V_S = 19 mV, I_T = 2 nA). The red curve indicates the fitting results of the Altsuler-Aronov effect.

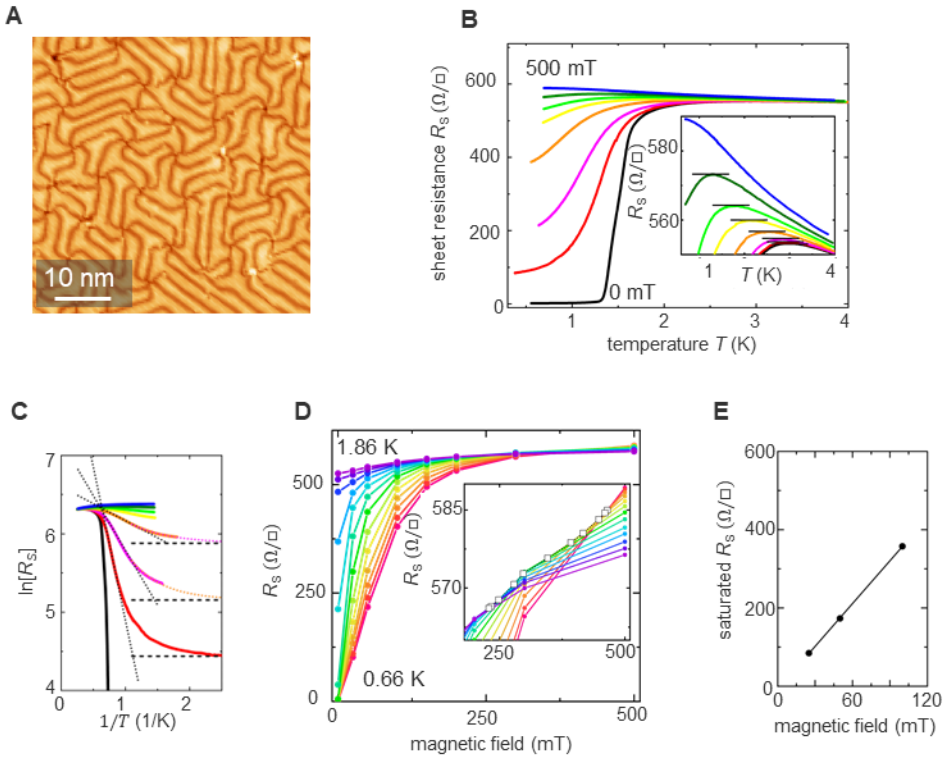

Fig. S4 Transport and STM results of the flat sample under out-of-plane magnetic fields. (**A**) STM image of the flat Si(111)-SIC-Pb sample (V_S = 1.6 V, I_T = 20 pA). (B) Temperature dependence of the sheet resistance of the flat sample under out-of-plane magnetic fields of 0, 25, 50, 100, 150, 200, 300, and 500 mT. The inset shows a magnified graph of the high-resistance region. (**C**) Arrhenius plots of (**B**). The dashed lines indicate the residual resistance at low temperatures owing to the anomalous metallic state, and the dotted lines indicate the fitting lines using the thermally assisted flux flow (TAFF) model. The dotted curves show exponential fitting. (**D**) R_S-B curves taken at various temperature, in 0.1 K steps from 0.66 K to 1.86 K. (**E**) Saturated resistance as a function of the magnetic field.

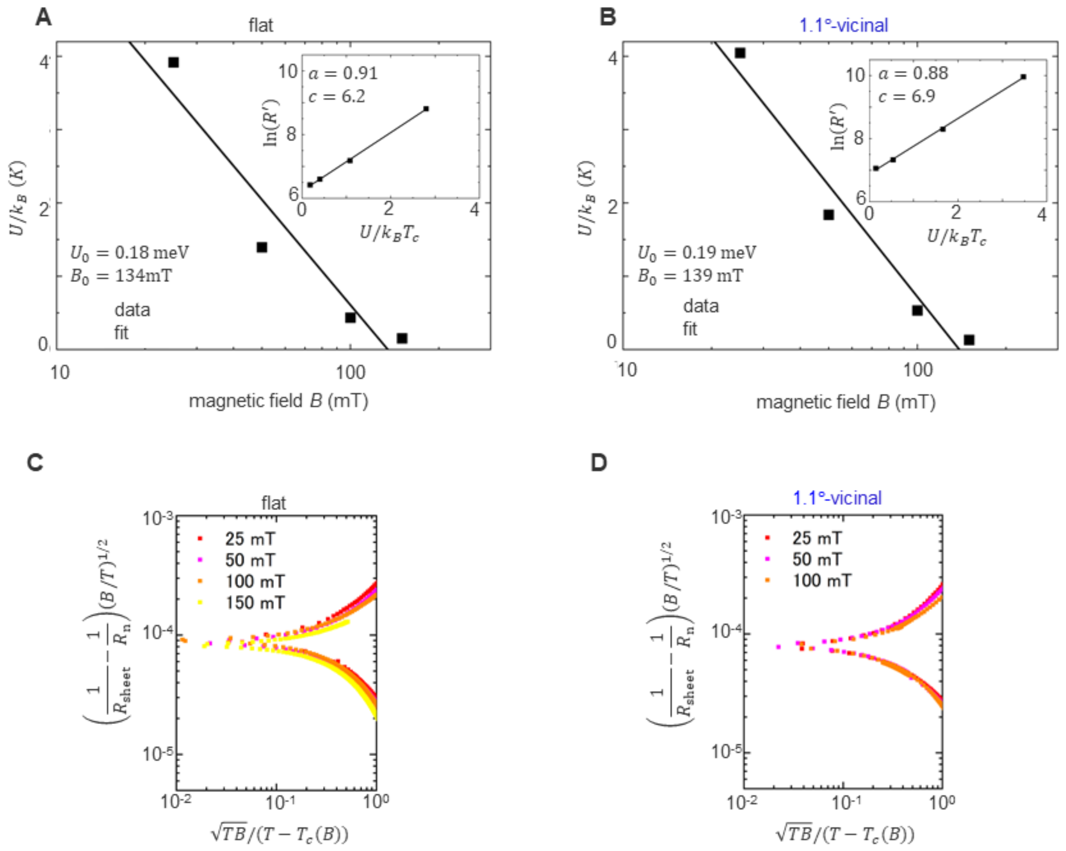

Fig. S5 Data analysis of results obtained from transport measurements. (**A** and **B**) Activation energies $U(B)/k_B$ of the (**A**) flat and (**B**) vicinal samples. The lines show the fitting results obtained using the equation $U(B) = U_0\ln(B_0/B)$. The insets show plots presenting the relationship between ln R’ and $U(B)/k_BT_c$. The solid lines show a linear fit, $\ln R' = aU(B)/k_BT_c + c$, where a and c are the fitting parameters. (**C** and **D**) Ullah-Dorsey (UD) scaling of fluctuation resistance using the data in Fig. 2A and Fig. S4B.

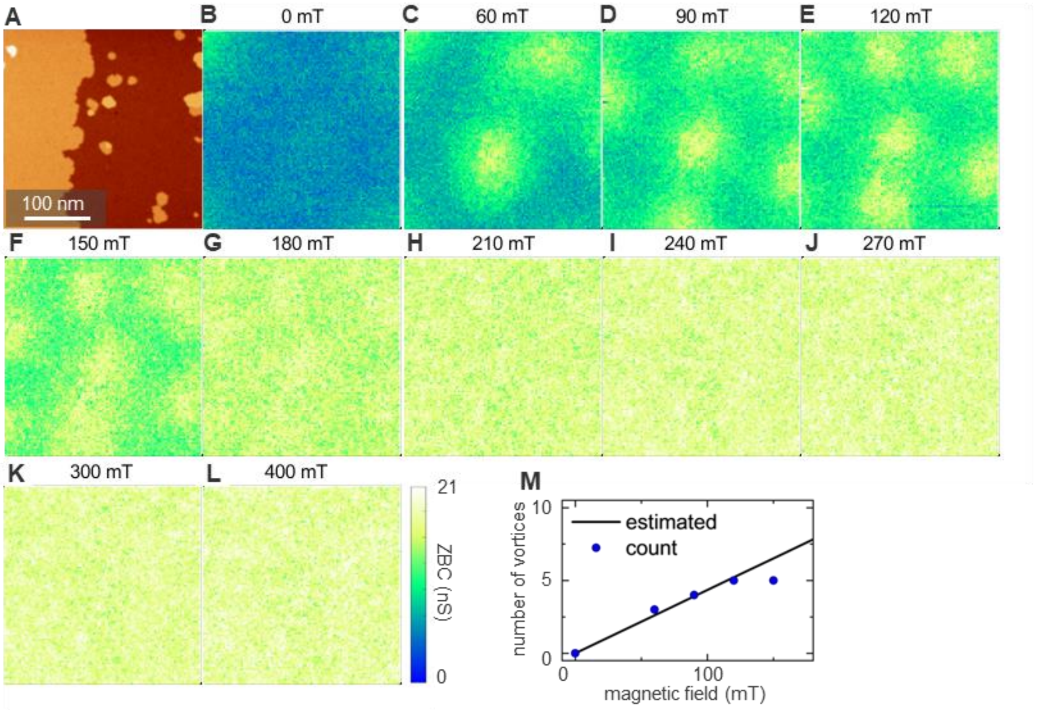

Fig. S6 STS measurements of the flat sample under out-of-plane magnetic fields. (**A**) STM image of the flat SIC sample. (**B** to **L**) Normalized ZBC maps under various magnetic fields taken in the same area as (**A**) (V_S = 9.2 mV, I_T = 200 pA). (**M**) The number of vortices as a function of the magnetic field.

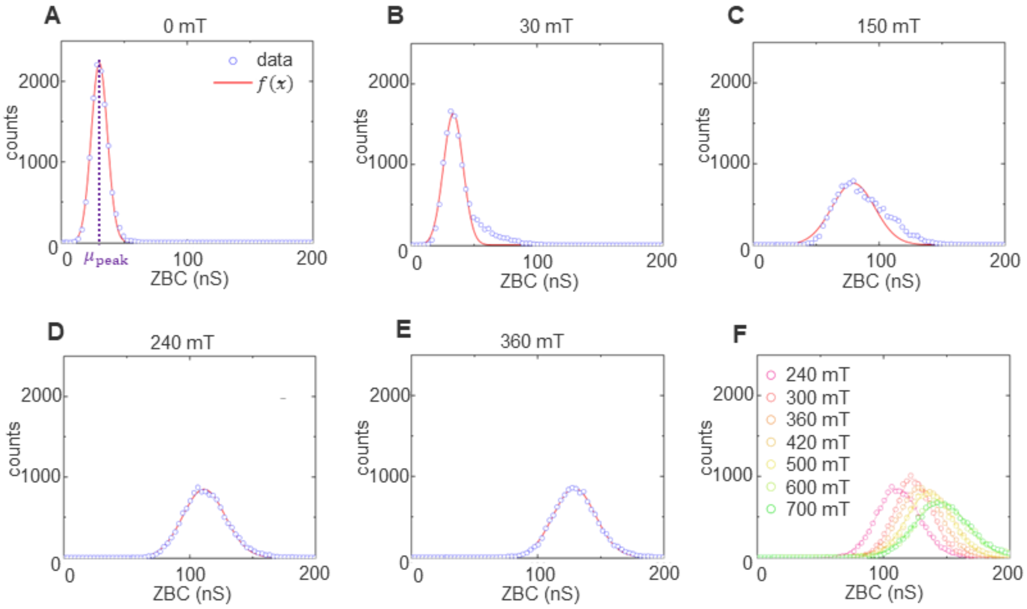

Fig. S7 Histograms of the ZBC values of the 1.1°-vicinal sample. (**A**) 0 mT (**B**) 30 mT (**C**) 150 mT (**D**) 240 mT (**E**) 360 mT (**F**) 240, 300, 360, 420, 500, 600 and 700 mT.

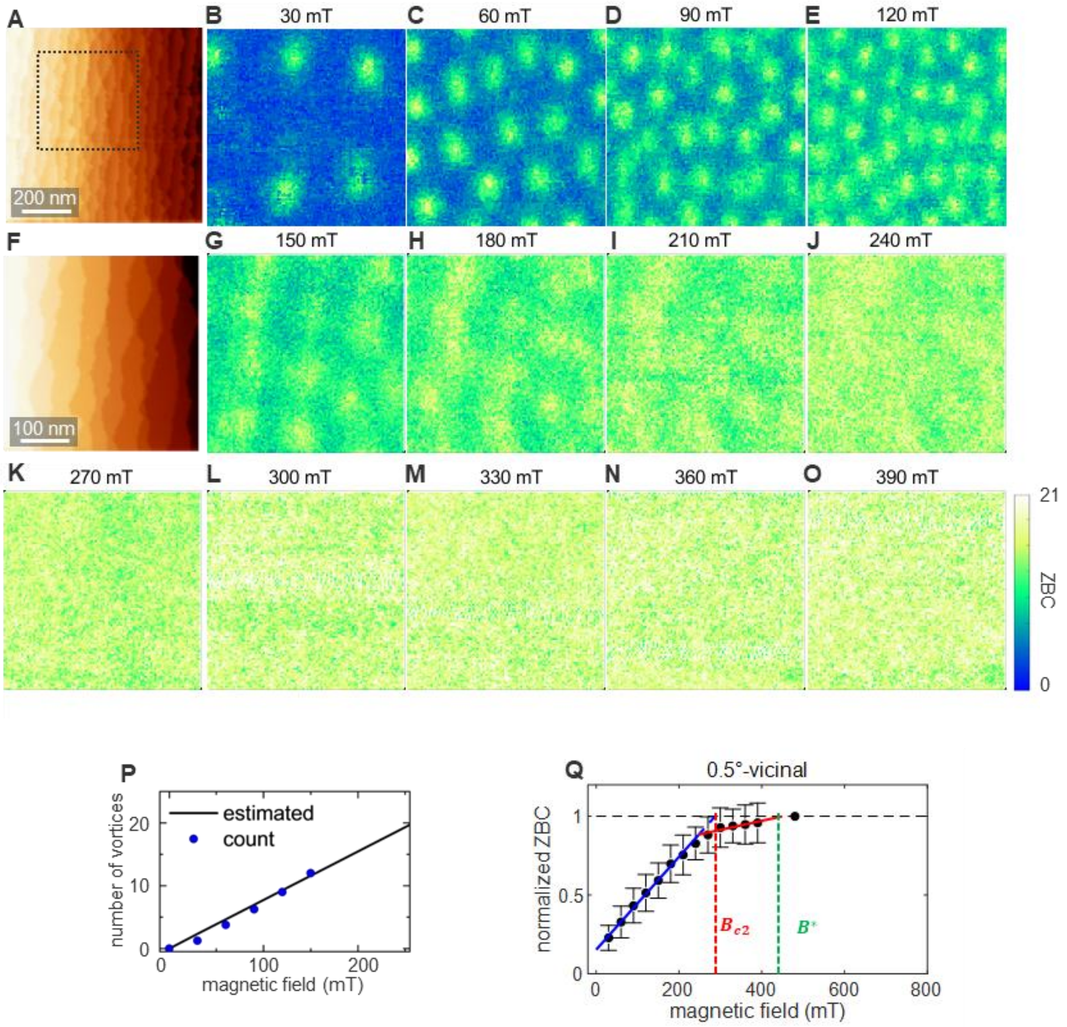

Fig. S8 STS measurements of a 0.5°-tilted vicinal sample under out-of-plane magnetic fields. (**A** and **F**) STM images taken on a SIC phase formed on a 0.5°-tilted vicinal substrate. (**F**) is the zoomed area indicating by dashed square in (**A**). (**B** to **E** and **G** to **O**) ZBC maps under various magnetic fields taken on the same area as (**A** and **F**), respectively (V_S = 10 mV, I_T = 200 pA). (**P** and **Q**) Evolution of the number of vortices and the peak ZBC values. Here, ZBC was normalized to the value measured at 480 mT (17 nS). We obtain B_{c2} = 287 mT and B^* = 449 mT.

REFERENCES

1. G. Logvenov, A. Gozar, I. Bozovic, High-temperature superconductivity in a single copper-oxygen plane. *Science* **326**, 699–702 (2009).

2. A. T. Bollinger, G. Dubuis, J. Yoon, D. Pavuna, J. Misewich, I. Bozovic, Superconductor-insulator transition in $La_{2-x}Sr_xCuO_4$ at the pair quantum resistance. *Nature* **472**, 458–460 (2011).

3. J. M. Lu, O. Zheliuk, I. Leermakers, N. F. Q. Yuan, U. Zeitler, K. T. Law, J. T. Ye, Evidence for two-dimensional Ising superconductivity in gated MoS_2. *Science* **350**, 1353–1357 (2015).

4. Y. Saito, Y. Kasahara, J. Ye, Y. Iwasa, T. Nojima, Metallic ground state in an ion-gated two-dimensional superconductor. *Science* **350**, 409–413 (2015).

5. Y. Saito, T. Nojima, Y. Iwasa, Quantum phase transitions in highly crystalline two-dimensional superconductors. *Nat. Commun.* **9**, 778 (2018).

6. X. Xi, Z. Wang, W. Zhao, J.-H. Park, K. T. Law, H. Berger, L. Forró, J. Shan, K. F. Mak, Ising pairing in superconducting $NbSe_2$ atomic layers. *Nat. Phys.* **12**, 139–143 (2015).

7. A. Tsen, B. Hunt, Y. Kim, Z. Yuan, S. Jia, R. Cava, J. Hone, P. Kim, C. Dean, A. Pasupathy, Nature of the quantum metal in a two-dimensional crystalline superconductor. *Nat. Phys.* **12**, 208–212 (2016).

8. S. Ichinokura, Y. Nakata, K. Sugawara, Y. Endo, A. Takayama, T. Takahashi, S. Hasegawa, Vortex-induced quantum metallicity in the mono-unit-layer superconductor $NbSe_2$. *Phys. Rev. B* **99**, 220501 (2019).

9. T. Zhang, P. Cheng, W.-J. Li, Y.-J. Sun, G. Wang, X.-G. Zhu, K. He, L. Wang, X. Ma, X. Chen, Y. Wang, Y. Liu, H.-Q. Lin, J.-F. Jia, Q.-K. Xue, Superconductivity in one-atomic-layer metal films grown on Si(111). *Nat. Phys.* **6**, 104–108 (2010).

10. C. Brun, T. Cren, V. Cherkez, F. Debontridder, S. Pons, D. Fokin, M. Tringides, S. Bozhko, L. Ioffe, B. Altshuler, D. Roditchev, Remarkable effects of disorder on superconductivity of single atomic layers of lead on silicon. *Nat. Phys.* **10**, 444–450 (2014).

11. A. Stępniak, A. Leon Vanegas, M. Caminale, H. Oka, D. Sander, J. Kirschner, Atomic layer superconductivity. *Surf. Interface Anal.* **46**, 1262–1267 (2014).

12. S. Yoshizawa, H. Kim, T. Kawakami, Y. Nagai, T. Nakayama, X. Hu, Y. Hasegawa, T. Uchihashi, Imaging Josephson vortices on the surface superconductor Si(111)−($\sqrt{7}\times\sqrt{3}$)−In using a scanning tunneling microscope. *Phys. Rev. Lett.* **113**, 247004 (2014).

13. S. Yoshizawa, H. Kim, Y. Hasegawa, T. Uchihashi, Disorder-induced suppression of superconductivity in the Si (111)−($\sqrt{7}\times\sqrt{3}$)-In surface: Scanning tunneling microscopy study. *Phys. Rev. B* **92**, 041410 (2015).

14. S. Ichinokura, L. Bondarenko, A. Tupchaya, D. Gruznev, A. Zotov, A. Saranin, S. Hasegawa, Superconductivity in thallium double atomic layer and transition into an insulating phase intermediated by a quantum metal state. *2D Materials* **4**, 025020 (2017).

15. F. Oguro, Y. Sato, K. Asakawa, M. Haze, Y. Hasegawa, Enhanced critical magnetic field for monoatomic-layer superconductor by Josephson junction steps. *Phys. Rev. B* **103**, 085416 (2021).

16. Y. Sato, M. Haze, R. Nemoto, W. Qian, S. Yoshizawa, T. Uchihashi, Y. Hasegawa, Squeezed Abrikosov-Josephson vortex in atomic-layer Pb superconductors formed on vicinal Si(111) substrates. *Phys. Rev. Lett.* **130**, 106002 (2023).

17. D. S. Baranov, S. Vlaic, J. Baptista, E. Cofler, V. S. Stolyarov, D. Roditchev, S. Pons, Gold atoms promote macroscopic superconductivity in an atomic monolayer of Pb on Si (111). *Nano Lett.* **22**, 652–657 (2022).

18. D. J. Bishop, E. G. Spencer, R. C. Dynes, The metal-insulator transition in amorphous Nb:Si. *Solid-State Electron.* **28**, 73–79 (1985).

19. B. G. Orr, H. M. Jaeger, A. M. Goldman, Local superconductivity in ultrathin Sn films. *Phys. Rev. B* **32**, 7586–7589 (1985).

20. D. Haviland, Y. Liu, A. M. Goldman, Onset of superconductivity in the two-dimensional limit. *Phys. Rev. Lett.* **62**, 2180–2183 (1989).

21. A. F. Hebard, M. A. Paalanen, Magnetic-field-tuned superconductor-insulator transition in two-dimensional films. *Phys. Rev. Lett.* **65**, 927–930 (1990).

22. H. M. Jaeger, D. B. Haviland, B. G. Orr, A. M. Goldman, Onset of superconductivity in ultrathin granular metal films. *Phys. Rev. B* **40**, 182–196 (1989).

23. S. Okuma, T. Terashima, N. Kokubo, Anomalous magnetoresistance near the superconductor-insulator transition in ultrathin films of α-Mo_xSi_{1-x}. *Phys. Rev. B* **58**, 2816–2819 (1998).

24. N. Marković, C. Christiansen, A. M. Goldman, Thickness–magnetic field phase diagram at the superconductor-insulator transition in 2D. *Phys. Rev. Lett.* **81**, 5217–5220 (1998).

25. A. Frydman, The superconductor insulator transition in systems of ultrasmall grains. *Physica C* **391**, 189–195 (2003).

26. C. Christiansen, L. M. Hernandez, A. M. Goldman, Evidence of collective charge behavior in the insulating state of ultrathin films of superconducting metals. *Phys. Rev. Lett.* **88**, 037004 (2002).

27. T. I. Baturina, A. Y. Mironov, V. Vinokur, M. Baklanov, C. Strunk, Localized superconductivity in the quantum-critical region of the disorder-driven superconductor-insulator transition in TiN thin films. *Phys. Rev. Lett.* **99**, 257003 (2007).

28. C. Brun, T. Cren, D. Roditchev, Review of 2D superconductivity: The ultimate case of epitaxial monolayers. *Supercond. Sci. Technol.* **30**, 013003 (2017).

29. C. D. Chen, P. Delsing, D. B. Haviland, Y. Harada, T. Claeson, Scaling behavior of the magnetic-field-tuned superconductor-insulator transition in two-dimensional Josephson-junction arrays. *Phys. Rev. B* **51**, 15645–15648 (1995).

30. A. J. Rimberg, T. R. Ho, C. Kurdak, J. Clarke, Dissipation-driven superconductor-insulator transition in a two-dimensional Josephson-Junction array. *Phys. Rev. Lett.* **78**, 2632–2635 (1997).

31. M. P. Fisher, Quantum phase transitions in disordered two-dimensional superconductors. *Phys. Rev. Lett.* **65**, 923–926 (1990).

32. A. E. White, R. C. Dynes, J. P. Garno, Destruction of superconductivity in quench-condensed two-dimensional films. *Phys. Rev. B* **33**, 3549–3552 (1986).

33. A. Yazdani, A. Kapitulnik, Superconducting-insulating transition in two-dimensional a-MoGe thin films. *Phys. Rev. Lett.* **74**, 3037–3040 (1995).

34. D. Ephron, A. Yazdani, A. Kapitulnik, M. R. Beasley, Observation of quantum dissipation in the vortex state of a highly disordered superconducting thin film. *Phys. Rev. Lett.* **76**, 1529–1532 (1996).

35. T. Uchihashi, Two-dimensional superconductors with atomic-scale thickness. *Supercond. Sci. Technol.* **30**, 013002 (2017).

36. K. Ienaga, T. Hayashi, Y. Tamoto, S. Kaneko, S. Okuma, Quantum criticality inside the anomalous metallic state of a disordered superconducting thin film. *Phys. Rev. Lett.* **125**, 257001 (2020).

37. B. Sacépé, M. Feigel'man, T. M. Klapwijk, Quantum breakdown of superconductivity in low-dimensional materials. *Nat. Phys.* **16**, 734–746 (2020).

38. C. G. L. Bøttcher, F. Nichele, M. Kjaergaard, H. J. Suominen, J. Shabani, C. J. Palmstrøm, C. M. Marcus, Superconducting, insulating and anomalous metallic regimes in a gated two-dimensional semiconductor–superconductor array. *Nat. Phys.* **14**, 1138–1144 (2018).

39. C. H. Sharma, A. P. Surendran, S. S. Varma, M. Thalakulam, 2D superconductivity and vortex dynamics in 1T-MoS_2. *Commun. Phys.* **1**, 90 (2018).

40. C. Yang, Y. Liu, Y. Wang, L. Feng, Q. He, J. Sun, Y. Tang, C. Wu, J. Xiong, W. Zhang, X. Lin, H. Yao, H. Liu, G. Fernandes, J. Xu, J. M. Valles Jr, J. Wang, Y. Li, Intermediate bosonic metallic state in the superconductor-insulator transition. *Science* **366**, 1505–1509 (2019).

41. Y. Xing, P. Yang, J. Ge, J. Yan, J. Luo, H. Ji, Z. Yang, Y. Li, Z. Wang, Y. Liu, F. Yang, P. Qiu, C. Xi, M. Tian, Y. Liu, X. Lin, J. Wang, Extrinsic and intrinsic anomalous metallic states in transition metal dichalcogenide Ising superconductors. *Nano Lett.* **21**, 7486–7494 (2021).

42. C. Yang, H. Liu, Y. Liu, J. Wang, D. Qiu, S. Wang, Y. Wang, Q. He, X. Li, P. Li, Y. Tang, J. Wang, X. C. Xie, J. M. Valles Jr, J. Xiong, Y. Li, Signatures of a strange metal in a bosonic system. *Nature* **601**, 205–210 (2022).

43. K. Ienaga, Y. Tamoto, M. Yoda, Y. Yoshimura, T. Ishigami, S. Okuma, Broadened quantum critical ground state in a disordered superconducting thin film. *Nat. Commun.* **15**, 2388 (2024).

44. E. Shimshoni, A. Auerbach, A. Kapitulnik, Transport through quantum melts. *Phys. Rev. Lett.* **80**, 3352–3355 (1998).

45. D. Das, S. Doniach, Existence of a Bose metal at $T = 0$. *Phys. Rev. B* **60**, 1261–1275 (1999).

46. A. Kapitulnik, N. Mason, S. A. Kivelson, S. Chakravarty, Effects of dissipation on quantum phase transitions. *Phys. Rev. B* **63**, 125322 (2001).

47. D. Dalidovich, P. Phillips, Phase glass is a Bose metal: A new conducting state in two dimensions. *Phys. Rev. Lett.* **89**, 027001 (2002).

48. P. Phillips, D. Dalidovich, The elusive Bose metal. *Science* **302**, 243–247 (2003).

49. P. W. Phillips, Not just a phase. *Nat. Phys.* **12**, 206–207 (2016).

50. Y. Noat, V. Cherkez, C. Brun, T. Cren, C. Carbillet, F. Debontridder, K. Ilin, M. Siegel, A. Semenov, H.-W. Hübers, D. Roditchev, Unconventional superconductivity in ultrathin superconducting NbN films studied by scanning tunneling spectroscopy. *Phys. Rev. B* **88**, 014503 (2013).

51. I. Roy, R. Ganguly, H. Singh, P. Raychaudhuri, Robust pseudogap across the magnetic field driven superconductor to insulator-like transition in strongly disordered NbN films. *Eur. Phys. J. B.* **92**, 49 (2019).

52. S. Dutta, J. Jesudasan, P. Raychaudhuri, Magnetic field induced transition from a vortex liquid to Bose metal in ultrathina-MoGe thin film. *Phys. Rev. B* **105**, L140503 (2022).

53. K. Horikoshi, X. Tong, T. Nagao, S. Hasegawa, Structural phase transitions of Pb-adsorbed Si (111) surfaces at low temperatures. *Phys. Rev. B* **60**, 13287–13290 (1999).

54. M. Yamada, Y. Sato, M. Haze, Y. Hasegawa, Simple model for striped-incommensurate phase formed by Pb adsorption on Si(111). *e-J. Surf. Sci. Nanotechnol.* **23**, 199–206 (2025).

55. R. S. Thompson, Microwave, flux flow, and fluctuation resistance of dirty type-II superconductors. *Phys. Rev. B* **1**, 327–333 (1970).

56. A. Larkin, A. Varlamov, *Theory of Fluctuations in Superconductors* (Clarendon, 2005).

57. J. Homoth, M. Wenderoth, T. Druga, L. Winking, R. Ulbrich, C. Bobisch, B. Weyers, A. Bannani, E. Zubkov, A. Bernhart, M. R. Kaspers, R. Möller, Electronic transport on the nanoscale: ballistic transmission and Ohm's law. *Nano Lett.* **9**, 1588–1592 (2009).

58. I. Matsuda, M. Ueno, T. Hirahara, R. Hobara, H. Morikawa, C. Liu, S. Hasegawa, Electrical resistance of a monatomic step on a crystal surface. *Phys. Rev. Lett.* **93**, 236801 (2004).

59. C. Tegenkamp, Z. Kallassy, H. Pfnur, H. L. Gunter, V. Zielasek, M. Henzler, Switching between one and two dimensions: Conductivity of Pb-induced chain structures on Si(557). *Phys. Rev. Lett.* **95**, 176804 (2005).

60. V. L. Berezinskii, Destruction of long-range order in one-dimensional and two-dimensional systems possessing a continuous symmetry group. II. Quantum systems. *Sov. Phys. JETP* **34**, 610–616 (1972).

61. J. M. Kosterlitz, D. J. Thouless, Ordering, metastability and phase transitions in two-dimensional systems. *J. Phys. C Solid State Phys.* **6**, 1181–1203 (1973).

62. R. C. Dynes, V. Narayanamurti, J. P. Garno, Direct measurement of quasiparticle-lifetime broadening in a strong-coupled superconductor. *Phys. Rev. Lett.* **41**, 1509–1512 (1978).

63. B. L. Altshuler, A. G. Aronov, Zero bias anomaly in tunnel resistance and electron-electron interaction. *Solid State Commun.* **30**, 115–117 (1979).

64. B. L. Altshuler, A. G. Aronov, P. A. Lee, Interaction effects in disordered Fermi systems in two dimensions. *Phys. Rev. Lett.* **44**, 1288–1291 (1980).

65. C. Carbillet, V. Cherkez, M. Skvortsov, M. Feigel'Man, F. Debontridder, L. Ioffe, V. Stolyarov, K. Ilin, M. Siegel, C. Noûs, Spectroscopic evidence for strong correlations between local superconducting gap and local Altshuler-Aronov density of states suppression in ultrathin NbN films. *Phys. Rev. B* **102**, 024504 (2020).

66. B. Sacépé, T. Dubouchet, C. Chapelier, M. Sanquer, M. Ovadia, D. Shahar, M. Feigel'man, L. Ioffe, Localization of preformed Cooper pairs in disordered superconductors. *Nat. Phys.* **7**, 239–244 (2011).

67. P. H. Kes, J. Aarts, J. van den Berg, C. J. van der Beek, J. A. Mydosh, Thermally assisted flux flow at small driving forces. *Supercond. Sci. Technol.* **1**, 242–248 (1989).

68. I. Guillamón, H. Suderow, A. Fernández-Pacheco, J. Sesé, R. Córdoba, J. M. De Teresa, M. R. Ibarra, S. Vieira, Direct observation of melting in a two-dimensional superconducting vortex lattice. *Nat. Phys.* **5**, 651–655 (2009).

69. J. Bardeen, M. J. Stephen, Theory of the motion of vortices in superconductors. *Phys. Rev.* **140**, A1197–A1207 (1965).

70. I. Tamir, A. Benyamini, E. J. Telford, F. Gorniaczyk, A. Doron, T. Levinson, D. Wang, F. Gay, B. Sacépé, J. Hone, K. Watanabe, T. Taniguchi, C. R. Dean, A. N. Pasupathy, D. Shahar, Sensitivity of the superconducting state in thin films. *Sci. Adv.* **5**, eaau3826 (2019).

71. A. Banerjee, A. Mohapatra, R. Ganesan, P. S. A. Kumar, Restoring superconductivity in the quantum metal phase of $NbSe_2$ using dissipative coupling. *Nano Lett.* **19**, 1625–1631 (2019).

72. S. Dutta, I. Roy, S. Mandal, J. Jesudasan, V. Bagwe, P. Raychaudhuri, Extreme sensitivity of the vortex state in a-MoGe films to radio-frequency electromagnetic perturbation. *Phys. Rev. B* **100**, 214518 (2019).

73. S. Ullah, A. T. Dorsey, Critical fluctuations in high-temperature superconductors and the Ettingshausen effect. *Phys. Rev. Lett.* **65**, 2066–2069 (1990).

74. N. Werthamer, E. Helfand, P. Hohenberg, Temperature and purity dependence of the superconducting critical field, H_{c2}. III. Electron spin and spin-orbit effects. *Phys. Rev.* **147**, 295–302 (1966).

75. Y. Xing, H.-M. Zhang, H.-L. Fu, H. Liu, Y. Sun, J.-P. Peng, F. Wang, X. Lin, X.-C. Ma, Q.-K. Xue, J. Wang, X. C. Xie, Quantum Griffiths singularity of superconductor-metal transition in Ga thin films. *Science* **350**, 542–545 (2015).

76. S. Okuma, S. Togo, M. Morita, Enhancement of the quantum-liquid phase by increased resistivity in thick a-Mo_xSi_{1-x} films. *Phys. Rev. Lett.* **91**, 067001 (2003).

77. Y. Dubi, Y. Meir, Y. Avishai, Nature of the superconductor-insulator transition in disordered superconductors. *Nature* **449**, 876–880 (2007).

78. S. Yoshizawa, T. Kobayashi, Y. Nakata, K. Yaji, K. Yokota, F. Komori, S. Shin, K. Sakamoto, T. Uchihashi, Atomic-layer Rashba-type superconductor protected by dynamic spin-momentum locking. *Nat. Commun.* **12**, 1462 (2021).

79. N. D. Mermin, H. Wagner, Absence of ferromagnetism or antiferromagnetism in one- or two-dimensional isotropic Heisenberg models. *Phys. Rev. Lett.* **17**, 1133–1136 (1966).

80. S. Coleman, There are no Goldstone bosons in two dimensions. *Commun. Math. Phys.* **31**, 259–264 (1973).

81. B. I. Halperin, D. R. Nelson, Resistive transition in superconducting films. *J. Low Temp. Phys.* **36**, 599–616, (1979).

82. M. R. Beasley, J. E. Mooij, T. P. Orlando, Possibility of vortex-antivortex pair dissociation in two-dimensional superconductors. *Phys. Rev. Lett.* **42**, 1165–1168, (1979).

83. M. V. Feigel'man, V. B. Geshkenbein, A. I. Larkin, Pinning and creep in layered superconductors. *Physica C* **167**, 177–187 (1990).
